# Development and validation of a novel pseudogene pair-based prognostic signature for prediction of overall survival in patients with hepatocellular carcinoma

**DOI:** 10.1186/s12885-020-07391-2

**Published:** 2020-09-16

**Authors:** Yajuan Du, Ying Gao

**Affiliations:** 1 Department of structural heart disease, the First Affiliated Hospital of Xi’an Jiaotong University, No.277, Yanta West Road, Xi’an, 710061 Shaanxi People’s Republic of China; 2 Department of Radiotherapy Oncology, the First Affiliated Hospital of Xi’an Jiaotong University, Xi’an, 710061 China

**Keywords:** Pseudogene pairs, Hepatocellular carcinoma, Survival, Signature

## Abstract

**Background:**

There is growing evidence that pseudogenes may serve as prognostic biomarkers in several cancers. The present study was designed to develop and validate an accurate and robust pseudogene pairs-based signature for the prognosis of hepatocellular carcinoma (HCC).

**Methods:**

RNA-sequencing data from 374 HCC patients with clinical follow-up information were obtained from the Cancer Genome Atlas (TCGA) database and used in this study. Survival-related pseudogene pairs were identified, and a signature model was constructed by Cox regression analysis (univariate and least absolute shrinkage and selection operator). All individuals were classified into high- and low-risk groups based on the optimal cutoff. Subgroups analysis of the novel signature was conducted and validated in an independent cohort. Pearson correlation analyses were carried out between the included pseudogenes and the protein-coding genes based on their expression levels. Enrichment analysis was performed to predict the possible role of the pseudogenes identified in the signature.

**Results:**

A 19-pseudogene pair signature, which included 21 pseudogenes, was established. Patients in high-risk group demonstrated an increased the risk of adverse prognosis in the TCGA cohort and the external cohort (all *P* < 0.001). The novel pseudogene signature was independent of other conventional clinical variables used for survival prediction in HCC patients in the two cohorts revealed by the multivariate Cox regression analysis (all *P* < 0.001). Subgroup analysis further demonstrated the diagnostic value of the signature across different stages, grades, sexes, and age groups. The C-index of the prognostic signature was 0.761, which was not only higher than that of several previous risk models but was also much higher than that of a single age, sex, grade, and stage risk model. Furthermore, functional analysis revealed that the potential biological mechanisms mediated by these pseudogenes are primarily involved in cytokine receptor activity, T cell receptor signaling, chemokine signaling, NF-κB signaling, PD-L1 expression, and the PD-1 checkpoint pathway in cancer.

**Conclusion:**

The novel proposed and validated pseudogene pair-based signature may serve as a valuable independent prognostic predictor for predicting survival of patients with HCC.

## Background

Hepatocellular carcinoma (HCC) is the most prevalent subtype of hepatic malignancies worldwide, accounting for 90% of primary liver cancers [1]. HCC is particularly prevalent in developing countries, particularly in East Asia and sub-Saharan Africa when compared with developed countries [2, 3]. Previous epidemiological studies have reported there to be approximately 250,000 new subjects and approximately 500,000 to 600,000 deaths due to HCC annually [1]. Despite the rapid advances in imaging techniques, surgical resection, and comprehensive therapy to treat HCC in recent years, the 5-year survival rate of HCC patients remains poor [4]. Therefore, it is necessary to uncover novel prognostic signatures that may identify groups of patients with a high risk of poor survival.

Pseudogenes are non-coding genes similar to their corresponding homologous protein-coding genes and long been considered ‘gene fossils’ or ‘junk genes’ because they do not encode functional proteins due to different kinds of mutations in the coding sequences [5]. In recent years, accumulating evidence has overwhelmingly revealed that individual pseudogenes involve in multiple human diseases including malignancy [6]. Multiple tumor-related pseudogenes have been confirmed as predictors for both diagnosis and prognosis. For example, the pseudogene DUXAP10 was found to be upregulated in several kinds of malignancies and could serve as a novel biomarker with high diagnostic and prognostic value for many cancers [7]. In HCC, high expression of the pseudogene ANXA2P2 has been found to be related to a worse prognosis. ANXA2P2 could be a novel predictive factor for evaluating the risk of recurrence or metastasis in HCC patients [8]. However, the molecular characteristics of pseudogene interactions and the prognostic value of pseudogenes in HCC have not been comprehensively explored.

Numerous studies have established mRNA expression profile-based signatures for outcome prediction in HCC patients [9–14]. However, these models have been failed to utilize clinically due to the diversity of data types, batch effects, and subsequent normalization of expression data, which poses a daunting obstacles for data processing given the possible biological heterogeneity among various data series and technical differences across different platforms [15]. Recently, a novel algorithm according to the relative orders of gene expression levels was established to remove the disadvantages of mRNA/miRNA expression normalization and scaling and has demonstrated robust results in previous studies [16, 17].

In this study, we identified 19 pseudogene-pairs based on univariate and LASSO regression analyses, and established a risk score model to predict the outcome of patients with HCC. Time-dependent receiver operating characteristic (ROC) curves were used to investigate the model’s performance in predicting the 1-, 3-, and 5-year overall survival (OS) of patients with HCC in two cohorts. Further, subgroup analysis was implemented to explore the prognostic performance of the signature in different stages, grades, sexes, and age groups. The C-index of the prognostic signature was compared with several established risk models. Pearson correlation analyses were done between the included pseudogenes and protein-coding genes based on their expression levels. Subsequently, we explored the biological functions and possible signaling pathways associated with the identified pseudogenes in the risk signature.

## Methods

### Data sources and pseudogene acquisition

The most current 13,600 pseudogenes were searched from the HUGO Gene Nomenclature Committee (HGNC, https://www.genenames.org/download/statistics-and-files/). RNA-sequencing (RNA-seq) data from 374 HCC patients and 50 normal controls with corresponding clinical follow-up information (370 with complete follow-up clinical data) was screened out from the Cancer Genome Atlas (TCGA) database. Pseudogene expression levels were determined using the GENCODE project (http://www.gencodegenes.org) annotation by repurposing the probes in the RNA-seq expression profiles. Additionally, mRNA expression matrix and the clinical follow-up information for 240 patients with primary HCC (231 with complete follow-up information) and 202 normal controls were downloaded from the International Cancer Genome Consortium database (ICGC, https://dcc.icgc.org/, LIRI-JP) to validate the model externally. The probe IDs were changed to their gene symbols based on their annotation files without further standardization. For more than one probes corresponding to the same gene symbol, the probe average was calculated as the final expression value of gene. Patient ID numbers were matched with their gene expression profiles and follow-up data. The mRNA expression matrix of the shared pseudogenes was extracted from these two publicly available datasets.

### Establishment of pseudogene pair-based prognostic signature

We first filtered out pseudogenes with imbalanced distribution or fairly little mutations [determined by median absolute deviation (MAD) < 0.5] across all samples in both cohorts [16]. Each pseudogene pair was analyzed by a pairwise comparison of pseudogene expression relative levels in a specific patient to obtain the score for per pseudogene pair. When the expression level of the first pseudogene more than the second pseudogene in a given pseudogene pair, the output value of the pseudogene pair was 1 and 0 for the different order, according to the proposed algorithm [16, 17]. Finally, 222 shared pseudogene pairs across two datasets were included. To explore the potential pseudogene pairs affecting the prognosis of HCC patients, univariate Cox regression analysis was used to identify the correlation between pseudogene pair expression and OS, with *P* < 0.05 being deemed statistically significant. Candidate factors were further screened by LASSO regression to yield the optimal informative but parsimonious model with 1000 iterations. Subsequently, a prognostic signature risk score was constructed according to the expression level of prognostic pseudogene pairs, weighted by the regression coefficient originated in the LASSO algorithm. Using the cutoff of the risk score generated by time-dependent ROC at 1 year for OS, all individuals were categorized into high- and low-risk groups.

### Validation of the prognostic performance of the pseudogene pair model

Kaplan-Meier analysis along with a log-rank test was applied to compare the survival differences of the two risk groups. Time-dependent ROC curve analysis for OS was carried out to determine the predictive power of the model. Univariate Cox regression was performed to determine potential prognostic variables, and multivariate Cox analysis was perform to verify the effect of the risk score model on prognosis and other clinical factors. Hazard ratios (HRs) and their 95% confidence intervals (CIs) were estimated.

### Comparison with other clinicopathological features and the novel prognostic model

To compare the effectiveness of the novel prognostic model with available clinicopathological factors and the recently built prognostic models, a comparison was implemented using the rcorrp.cens package in Hmisc in R and evaluated by C-index with 1000 bootstrap resamples.

### Identification and enrichment analysis of pseudogene-related protein-coding genes

The Pearson correlation coefficients (|Pearson correlation coefficient| > 0.6 and *P*-value < 0.001) between the final identified pseudogenes and protein-coding genes were measured to detect their co-expression associations [18]. Gene Ontology (GO) functional enrichment analysis as well as Kyoto Encyclopedia of Genes and Genomes (KEGG) pathway enrichment analyses were also conducted utilizing the clusterProfiler package to investigate the biological function and pathways involving numerous genes [19].

### Statistical analysis

Survival curves were generated using the Kaplan–Meier method along with the log-rank test. Receiver operating characteristic (ROC) curves were generated using the R package “survivalROC”. The area under the curve (AUC) value obtained from the ROC curve was used to explore the diagnostic effectiveness of signature risk score in discriminating HCC tissues from normal tissues in two cohorts. Multivariate analyses were carried out utilizing the Cox proportional hazards regression model. A *P*-value less than 0.05 was considered significant.

## Results

### Establishing the pseudogene pair-based signature

**Fig. 1:**
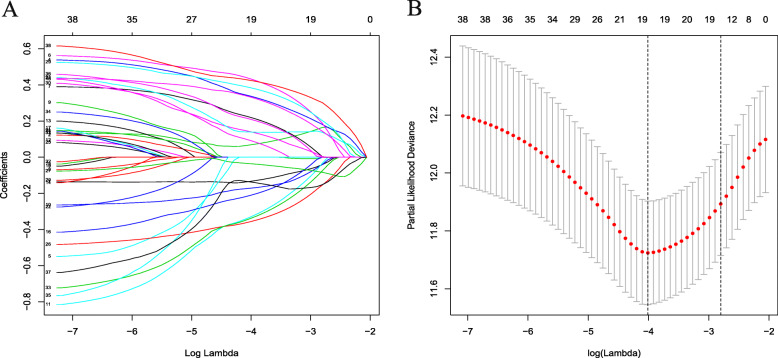
Predictor selection by LASSO algorithm. **a**: Parameter filter by LASSO regress algorithm used five-fold cross-validation by through minimum criteria; **b**: Optimal feature selection based on LASSO coefficient profile plot of 19 pseudogene pairs

The follow-up clinical information of patients in the two cohorts were shown in Table 1. A total of 222 pseudogene pairs were identified from 36 shared pseudogenes in the TCGA cohort after filtering by MAD > 0.5 as mentioned above. Univariate Cox regression analysis was carried out for the 222 pseudogene pairs to reveal 38 pseudogene pairs presenting significant prognostic potential (*P* < 0.05). Next, we performed LASSO Cox regression algorithm to reduce the number of pseudogene pairs in the risk model. After 1000 iterations, 19 pseudogene pairs were obtained and used to build a prognostic risk signature (Fig. 1). The risk signature consisted of 21 unique pseudogenes (Table 2).

**Table 1:** Clinical data of patients in the TCGA and the ICGC validation cohort

Variables	Subgroups	TCGA (***N*** = 370)	ICGC(***N*** = 231)
Age	< 60	169	44
	> = 60	201	187
Sex	Male	249	179
	Female	121	62
Stage	I	171	36
	II	85	104
	III	85	72
	IV	5	19
	NA	24	0
Grade	I	55	–
	II	177	–
	III	121	–
	IV	12	–
	NA	5	–
Survival status	Dead	130	42
	Living	240	189
Vascular invasion	Positive	108	–
	Negative	206	–
	NA	56	–
Family history	Positive	112	73
	Negative	207	143
	NA	51	15
Prior malignancy	Positive	–	29
	Negative	–	202
	NA	–	0

**Table 2:** Information on the 19 pseudogene pairs and the coefficient obtained from the least absolute shrinkage and selection operator (LASSO) regression analysis

Genepair1	Full name	Genepair2	Full name	Coef
ABCC6P2	ATP binding cassette subfamily C member 6 pseudogene 2	DSTNP2	DSTN pseudogene 2	−0.133577486
ANXA2P2	annexin A2 pseudogene 2	AZGP1P1	AZGP1 pseudogene 1	0.06815618
ANXA2P2	annexin A2 pseudogene 2	HLA-J	major histocompatibility complex, class I, J	0.337854755
AQP7P1	aquaporin 7 pseudogene 1	HLA-J	major histocompatibility complex, class I, J	0.433464122
AQP7P1	aquaporin 7 pseudogene 1	MT1DP	metallothionein 1D, pseudogene	0.220401079
AZGP1P1	AZGP1 pseudogene 1	CYP21A1P	cytochrome P450 family 21 subfamily A member 1, pseudogene	−0.171662304
AZGP1P1	AZGP1 pseudogene 1	GGTA1P	glycoprotein alpha-galactosyltransferase 1, pseudogene	−0.330772998
C3P1	complement component 3 precursor pseudogene	MT1L	metallothionein 1 L, pseudogene	−0.211202632
CA5BP1	carbonic anhydrase 5B pseudogene 1	LPAL2	lipoprotein(a) like 2, pseudogene	0.140891921
DSTNP2	DSTN pseudogene 2	PLGLA	plasminogen like A	0.139199981
DSTNP2	DSTN pseudogene 2	WASH3P	WASP family homolog 3, pseudogene	0.332685477
HLA-J	major histocompatibility complex, class I, J	MSTO2P	misato family member 2, pseudogene	−0.356768111
HLA-J	major histocompatibility complex, class I, J	RP9P	RP9 pseudogene	−0.035991571
HSPA7	heat shock protein family A (Hsp70) member 7 (pseudogene)	NAPSB	napsin B aspartic peptidase, pseudogene	0.384325838
LPAL2	lipoprotein(a) like 2, pseudogene	PLGLA	plasminogen like A	0.092279424
NAPSB	napsin B aspartic peptidase, pseudogene	NSUN5P1	NSUN5 pseudogene 1	−0.339252375
NUDT16P1	nudix hydrolase 16 pseudogene 1	PLGLA	plasminogen like A	0.20989673
PLGLA	plasminogen like A	RP9P	RP9 pseudogene	−0.137033874
RP9P	RP9 pseudogene	WASH3P	WASP family homolog 3, pseudogene	0.424813675

### Association between signature risk score and clinical characteristics

**Fig. 2:**
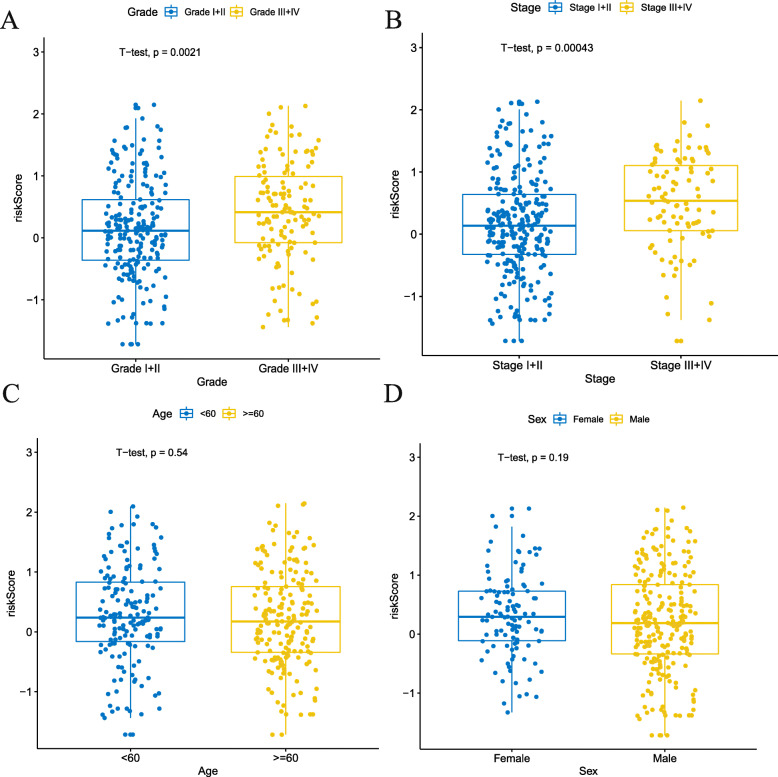
Association between the pseudogene pair-based signature risk score and clinical parameters in the TCGA cohort

To confirm the clinical value of the pseudogene pair-based signature risk score, the Chi-square test was applied to assess the association between the risk score and available clinical parameters. In the TCGA cohort, a higher risk score was revealed to be associated notably with grade (III + IV vs grade I + II, *P* = 0.0021; Fig. 2a) and stage (III + IV vs I + II, *P* = 0.00043; Fig. 2b). However, no significant difference was found in age (*P* = 0.0021; Fig. 2c) and gender (*P* = 0.0021; Fig. 2d).

### Validation and assessment of the established signature

**Fig. 3:**
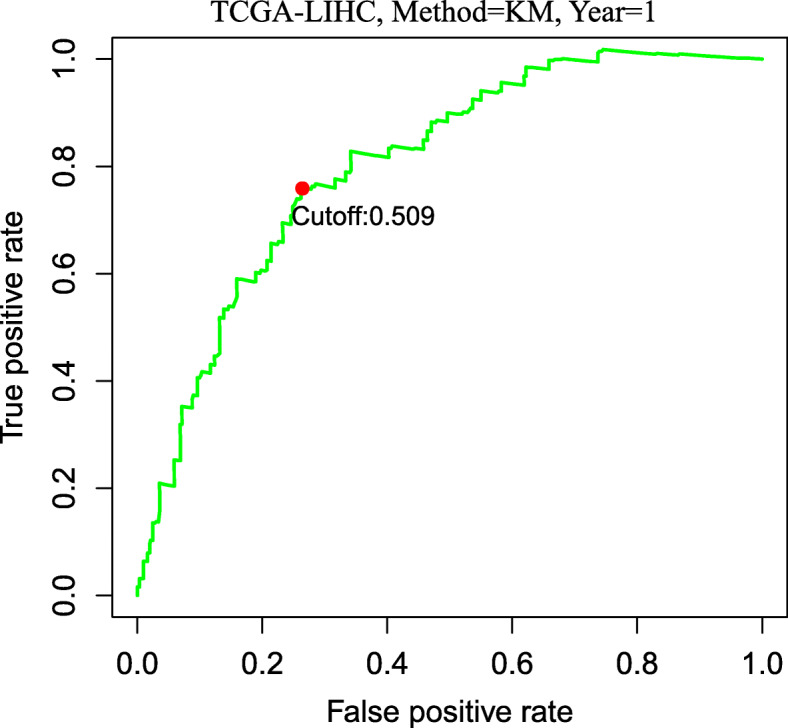
Time-dependent ROC curve analysis of the risk score. A cutoff point of risk score was identified as 0.509 to divide patients into two distinct groups in the TCGA cohort

**Fig. 4:**
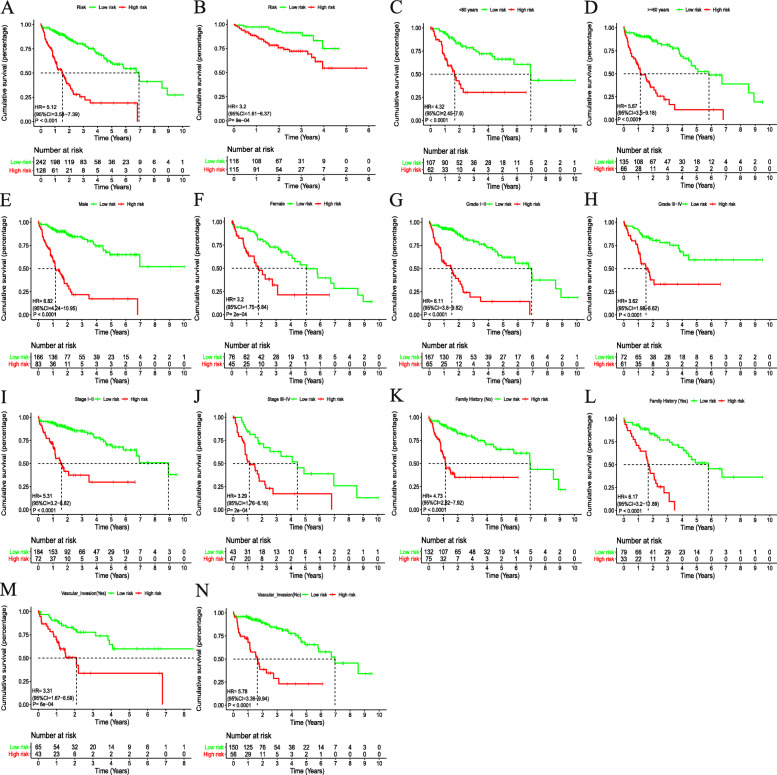
Kaplan-Meier survival curves for patients with HCC in two distinct groups. Survival cures in the TCGA cohort (**a**), ICGC dataset (**b**), and subgroup analysis with respect to age (**c**, **d**), gender (**e**, **f**), histological grade (**g**, **h**), American Joint Committee on Cancer stage (**i**, **j**), family history (**k**, **l**), and vascular invasion (**m**, **n**)

Next, the risk score of the novel signature for per patient were calculated in the TCGA cohort. The optimal cutoff score for classifying patients into high- or low-risk groups was determined as 0.509 employing time-dependent ROC curve analysis at 1 year for OS predication (Fig. 3). High-risk patients exhibited a worse prognosis than low-risk patients, as revealed by Kaplan-Meier and log-rank tests (HR: 5.12, 95% CI: 3.54.7.39, *P* < 0.001, Fig. 4a). Patients in high-risk group also had worse outcomes than low-risk patients in the ICGC cohort (HR = 3.2, 95%CI: 1.61–6.37, *P* < 0.001, Fig. 4b) using the same cutoff point as in the TCGA dataset.

To evaluate the prognostic performance of the signature in different subgroups, we investigated the relationship between clinical pathological factors and the prognostic signature using Kaplan-Meier and log-rank tests. As shown in Fig. 4c-n, the Kaplan–Meier curves illustrated that the signature was a robust prognostic predictor for patients with HCC grouped by sex (male or female), age (< 60 years or ≥ 60 years), family history (Yes or No),grade (grade I-II or grade III-IV), vascular invasion (Yes or No), and stage (stage I-II or stage III-IV). Multivariate Cox regression analyses were used to screen out the independent predictor in two cohorts. After adjusting for other clinical and pathological variables, the prognostic signature risk score was still an independent prognostic variable for OS in the TCGA cohort (HR = 3.416, 95%CI: 2.551–4.576; *P* < 0.001) and was validated in the ICGC cohort (HR = 1.902, 95%CI: 1.201–3.014, *P* = 0.006, Table 3).

**Table 3:** Univariate and multivariate analyses identified independent prognostic factors for overall survival of HCC in the TCGA and the ICGC cohorts

	Univariate analysis			Multivariate analysis		
	HR	95%CI	*P*-value	HR	95%CI	*P*-value
**TCGA cohort**						
Age	1.01	0.996–1.025	0.174	1.01	0.996–1.024	0.168
Sex	0.776	0.531–1.132	0.188	0.912	0.614–1.353	0.646
Grade	1.133	0.881–1.456	0.33	0.927	0.706–1.219	0.588
Stage	1.68	1.369–2.062	< 0.0001	1.33	1.070–1.654	0.01
riskScore	3.583	2.726–4.709	< 0.0001	3.416	2.551–4.576	< 0.0001
**ICGC cohort**						
Sex	0.515	0.270–0.982	0.044	0.42	0.215–0.819	0.011
Age	0.998	0.966–1.032	0.917	0.989	0.955–1.025	0.558
Stage	2.238	1.532–3.269	< 0.0001	2.16	1.459–3.198	0.0001
Prior malignancy	1.658	0.692–3.975	0.257	2.287	0.912–5.734	0.078
Cancer history	0.794	0.404–1.563	0.505	0.706	0.351–1.421	0.329
riskScore	2.337	1.490–3.664	0.0002	1.902	1.201–3.014	0.006

**Fig. 5:**
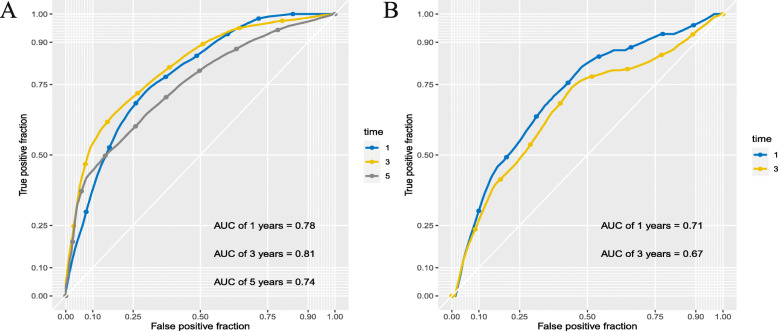
The ROC curve for 1-, 3- and 5-year overall survival prediction using the pseudogene pair-based prognostic. **a** TCGA cohort; **b** ICGC cohort

Furthermore, the AUC values of the prognostic model for the 1-, 3-, and 5-year survival rates prediction in the TCGA cohort were 0.78, 0.81, and 0.74, respectively, (Fig. 5a). This revealed the predictive performance of the prognostic signature to be quite promising. The AUC values for OS in the ICGC cohort at 1 year and 3 years were 0.71 and 0.67, respectively (Fig. 5b). These findings confirmed that the novel model accurately predicted the prognosis of patients with HCC.

**Fig. 6:**
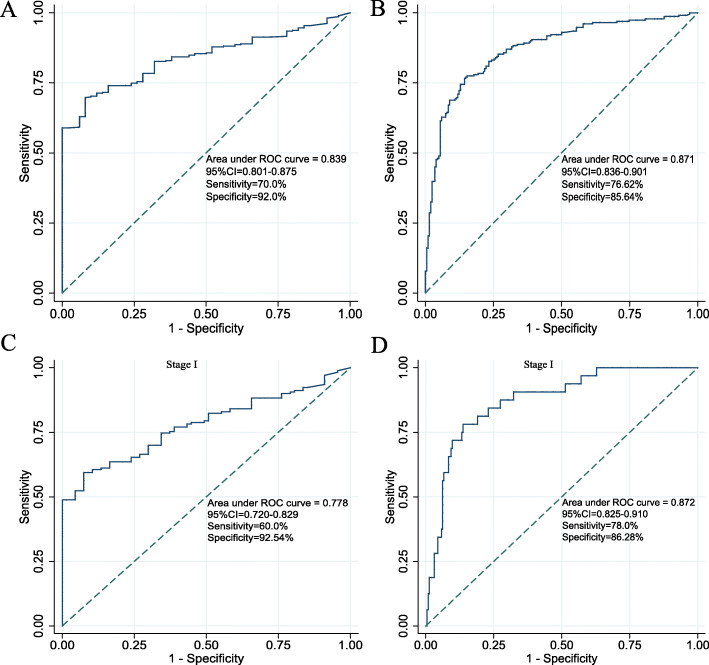
Diagnosis value of pseudogene pair-based signature risk score in HCC and normal controls. ROC in normal tissues and HCC samples in the TCGA cohort (**a**) and ICGC cohort (**b**). ROC for stage I samples and normal tissues in the TCGA cohort (**c**) and ICGC cohort (**d**)

To explore the diagnostic value of pseudogene pair-based signature, we generated a ROC curve using the risk score from 374 HCC patients and 50 healthy controls. The AUC was 0.839 (95%CI = 0.801–0.875; Fig. 6a), which was further confirmed in the ICGC cohort with an AUC of 0.871 (95%CI = 0.836–0.901; Fig. 6b). Subgroup analysis demonstrated the diagnostic value of signature risk score in early stage of HCC were robust with AUC value of 0.778 (95%CI = 0.720–0.829; Fig. 6c) for stage I disease in the TCGA cohort. The diagnostic power was confirmed in the ICGC cohort with an AUC of 0.872 (95%CI = 0.825–0.910; Fig. 6d) for stage I disease. These demonstrated that the pseudogene pair-based signature risk score had an excellent diagnostic value in discriminating HCC from normal samples.

### Comparison with previous existed prognostic signatures

**Fig. 7:**
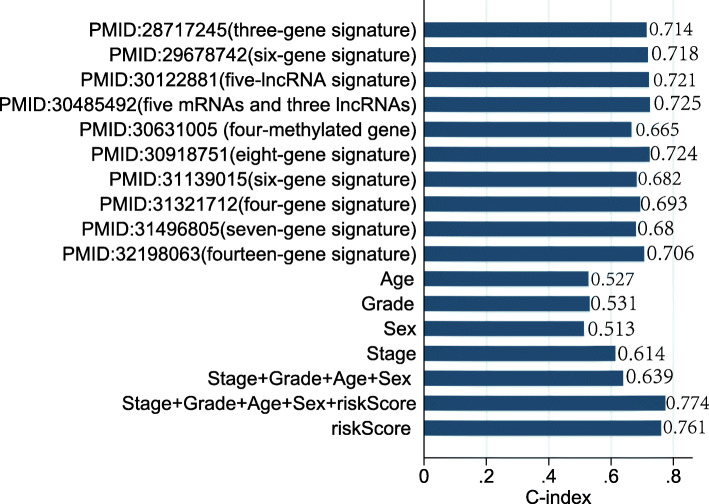
Comparison of C-index among the novel model, previously established prognostic signatures, and clinical features (age, sex, stage, grade, and their combination)

We compared our novel model with previous established prognostic signatures and confirmed the predictive performance and precision of the signature. Most importantly, the novel signature yielded a C-index of 0.761, which was higher than that of risk models based on single variable, which included age, grade, sex, stage as well as the merged models (all *P* < 0.05, Fig. 7). Furthermore, we also compared our model with recent existing signatures used to predict HCC survival. The C-index of our prognostic signature was larger than that of previous existed models (all *P* < 0.05). In addition, the C-index of the signature combined with other variables was 0.774. Thus, a combination of our prognostic signature and other variables should provide a more accurate prediction. Therefore, the novel prognostic signature was robust in predicting the prognosis of HCC patients.

### Functional analysis of co-expression genes

To further example the potential biological roles of the 21 unique pseudogenes identified, the protein-coding genes positively or negatively correlated with them (|Pearson correlation coefficient| > 0.6 and *P*-value < 0.001) were considered pseudogene-related protein-coding genes. A total of 842 genes were considered eligible for pathway enrichment. We conducted GO and KEGG enrichment analyses to uncover specific functional categories of the co-expressed genes. They were primarily involved in cytokine receptor activity, cytokine binding, chemokine receptor activity, C-C chemokine receptor activity, and chemokine binding (Table 4). KEGG pathway enrichment revealed that these genes were primarily involved in T cell receptor signaling, chemokine signaling, B cell receptor signaling, PD-L1 expression, NF-κB signaling, and the PD-1 checkpoint pathway in cancer (Table 4).

**Table 4:** GO functional and KEGG pathway enrichment analysis of pseudogenes-related protein-coding genes

ID	Description	***P*** value	P adjust
GO:0004896	cytokine receptor activity	1.64E-11	6.31E-09
GO:0001637	G protein-coupled chemoattractant receptor activity	4.05E-08	3.23E-06
GO:0004950	chemokine receptor activity	4.05E-08	3.23E-06
GO:0019955	cytokine binding	4.21E-08	3.23E-06
GO:0016493	C-C chemokine receptor activity	1.65E-07	9.05E-06
GO:0019957	C-C chemokine binding	2.54E-07	1.30E-05
GO:0019956	chemokine binding	3.92E-07	1.88E-05
GO:0023023	MHC protein complex binding	6.72E-07	3.04E-05
GO:0042287	MHC protein binding	1.33E-06	5.67E-05
GO:0032395	MHC class II receptor activity	2.54E-05	0.001027582
GO:0030246	carbohydrate binding	0.000143695	0.004598232
GO:0001608	G protein-coupled nucleotide receptor activity	0.000175825	0.005193614
GO:0045028	G protein-coupled purinergic nucleotide receptor activity	0.000175825	0.005193614
GO:0030695	GTPase regulator activity	0.000508907	0.012607749
KEGG:hsa04662	B cell receptor signaling pathway	4.67E-12	1.85E-10
KEGG:hsa04062	Chemokine signaling pathway	2.79E-09	4.07E-08
KEGG:hsa04660	T cell receptor signaling pathway	1.36E-07	1.45E-06
KEGG:hsa04650	Natural killer cell mediated cytotoxicity	3.92E-07	4.02E-06
KEGG:hsa04060	Cytokine-cytokine receptor interaction	1.07E-06	1.03E-05
KEGG:hsa04064	NF-kappa B signaling pathway	0.000577911	0.00390442
KEGG:hsa05235	PD-L1 expression and PD-1 checkpoint pathway in cancer	0.001387015	0.008934958
KEGG:hsa05231	Choline metabolism in cancer	0.008939182	0.047618336

## Discussion

HCC remains a major and growing global public health challenge. However, the molecular pathogenesis of HCC is not fully understood. Given the extensive heterogeneity of HCC, there is a need for more accurate individualized prognostic signatures. Recently, increasing evidence has demonstrated that abnormal expression of pseudogenes is involved in multiple diseases, including malignancy [6]. For example, in HCC, upregulation of the pseudogene RP11-564D11.3 has been found to be associated with adverse survival [20]. Numerous researches have built gene expression profile-based signatures for survival prediction in patients with HCC [9–14]. However, previous reports aiming to build a prognostic model have focused on mRNAs, lncRNAs, and miRNAs, neglecting pseudogenes as potential biomarkers in HCC. Therefore, the development of a robust pseudogene pair signature contributes to clinical decision-making for individualized treatment of HCC patients.

In this study, we established a novel 19-pseudogene pair signature that could successfully classify patients into two groups with different OS. We found that patients in high-risk group had a worse survival rate than patients in the low-risk group in both cohorts. Subgroup analysis by age, family history, sex, grade, vascular invasion, and stage yielded the same conclusion. We found the signature to be a stable prognostic predictor for patients with HCC. Multivariate analyses demonstrated that the risk score may be a clinically independent prognostic predictor for HCC. The AUC values of the prognostic model for OS prediction also present excellent predictive performance in both cohorts. The signature was reproducible and robust in the independent validation cohort, demonstrating its value and effectiveness. These conclusions confirmed that the novel model could offer an accurate survival prediction for patients with HCC. Moreover, the C-index of our signature was larger than that of established signatures. We employed a more comprehensive and novel approach to develop a robust prognostic signature for HCC and successfully validated it in the ICGC cohort. Therefore, this novel prognostic model is accurate, robust, and interpretable.

Although numerous prognostic models have been established for the prediction of HCC survival [9–14, 21, 22], these prognostic models have seldom been widely utilized clinically due to their need for proper data standardization across various expression profiles for further analysis [16, 17]. In this study, based on the relative orders of the mRNA expression, the signature was generated only by weight-pairwise comparison within a given sample without requiring for data normalization and can remove the batch effects between different platforms. Furthermore, the cutoff value derived from the risk score formula used in this study could be employed across multiple datasets, showing a great advantage when compared with previous models, and may be easily translated into clinical application. This novel algorithm has been validated to be accurate and robust in previous cancer-related reports [16, 17, 23, 24].

The identified pseudogene-related protein-coding genes were primarily involved in cytokine and chemokine receptor activity, and cancer-related pathways, such as T cell receptor signaling, NF-κB signaling, PD-L1 expression, and PD-1 checkpoint pathway in cancer. It has also been reported that IL-8 and IL-6, important chemokines, are involved in tumor angiogenesis, growth and metastasis, and can therefore act as vital chemokines for blood vessel formation in HCC [25–27]. Previous studies have confirmed that T cells gather in the blood of HCC patients, and tumor necrosis factor (TNF), which is involve in the T cell receptor signaling pathway, was downregulated in sorafenib-treated HCC patients, demonstrating that the T cell receptor signaling pathway may also be involved in HCC [28, 29]. A previous study has provided evidence for the inhibitory effect of PPARα on HCC via the NF-κB signaling pathway [30]. IGFBP2 can serve as a new therapeutic target that activates the NF-κB-ZEB1 signaling axis and contributes to HCC tumorigenesis [31]. Expression of immune checkpoint molecules, such as PD-1/PD-L1, has been confirmed in HCC [32]. Furthermore, in September 2017, the FDA has granted accelerated approval to PD-1 checkpoint inhibitors for the treatment of HCC patients [33]. Therefore, the novel established pseudogene pair signature could be associated with HCC-related biological pathways and the functional dysregulation could be well associated with the survival of HCC patients.

The signature based on the relative expression ordering exhibited no difficulty in clinical transformation and application. For future study, if the expression matrix of 21 pseudogenes was obtained, a 19-pseudogene pair signature was therefore constructed. Furthermore, the signature only involves pairwise comparison within a given sample without the requiring for data normalization and batch effects from different laboratories and platforms. Based on the cutoff value derived from the risk score formula, patients can be grouped into different groups, and their prognosis can be predicted. Thus, the prognostic model can act as an individualized, single-sample prediction of outcome of HCC and can be easily translated to clinical application.

This is the first research to establish a pseudogene pair-based prognostic signature in HCC. However, this study also has limitations. First, prospective research is suggested to validate the prognostic function of the pseudogene pair signature. Moreover, to better understand the functional role of these pseudogene pairs in HCC, experimental studies investigating these pseudogenes should be carried out.

## Conclusion

We developed and validated an accurate and novel robust pseudogene pair signature capable of accurately predicting the prognosis of HCC patients, with higher risk scores demonstrating adverse prognosis. The signature is reproducible and robust in an independent external cohort and outperforms other established signatures, demonstrating its value and effectiveness. Additionally, this signature could act as an encouraging independent prognostic predictor for HCC.

## Data Availability

All the data used in the study are obtained from the TCGA database (https://portal.gdc.cancer.gov/) and ICGC database (https://dcc.icgc.org/), which are opening and available to all.

## Abbreviations

TCGA: The Cancer Genome Atlas
HCC: Hepatocellular carcinoma
LASSO: Least absolute shrinkage and selection operator
ROC: Receiver operating characteristic
OS: Overall survival
GO: Gene Ontology
HR: Hazard ratios
C-index: Concordance index
CI: Confidence interval
AUC: Area under the curve
KEGG: Kyoto Encyclopedia of Genes and Genomes
